# Atorvastatina Atenua o Remodelamento Vascular em Camundongos com Síndrome Metabólica

**DOI:** 10.36660/abc.20200322

**Published:** 2021-06-10

**Authors:** Karine Ferreira da Silva Carvalho, Amanda Araújo Marques Ferreira, Nayara Carvalho Barbosa, Juliano Vilela Alves, Rafael Menezes da Costa

**Affiliations:** 1 Universidade Federal de Jataí Unidade Acadêmica de Ciências da Saúde JataíGO Brasil Universidade Federal de Jataí - Unidade Acadêmica de Ciências da Saúde, Jataí, GO - Brasil; 2 Faculdade de Medicina de Ribeirão Preto Universidade de São Paulo Departamento de Farmacologia Ribeirão PretoSP Brasil Faculdade de Medicina de Ribeirão Preto, Universidade de São Paulo - Departamento de Farmacologia, Ribeirão Preto, SP - Brasil

**Keywords:** Dislipidemias, Carbohidratos da Dieta, Síndrome Metabólica, Remodelamento Vascular, Atorvastatina, Inflamação, Camundongo

## Abstract

**Fundamento:**

A síndrome metabólica é caracterizada por um conjunto de comorbidades. Durante a síndrome, observam-se alterações estruturais no sistema cardiovascular, especialmente o remodelamento vascular. Uma das causas predisponentes para essas alterações é a inflamação crônica oriunda de mudanças na estrutura e composição do tecido adiposo perivascular. Atorvastatina é eficaz no tratamento das dislipidemias. No entanto, seus efeitos pleiotrópicos não são totalmente compreendidos. Supõe-se que, durante a síndrome metabólica, ocorre remodelamento vascular e que o tratamento com atorvastatina pode ser capaz de atenuar tal condição.

**Objetivos:**

Avaliar os efeitos do tratamento com atorvastatina sobre o remodelamento vascular em modelo experimental de síndrome metabólica.

**Métodos:**

Camundongos Swiss receberam dieta controle ou dieta hiperglicídica por 18 semanas. Após 14 semanas de dieta, os camundongos foram tratados com veículo ou atorvastatina (20mg/kg) durante 4 semanas. Foram avaliados o perfil nutricional e metabólico por testes bioquímicos; análise estrutural da artéria aorta por histologia e dosagem de citocinas por ensaio imunoenzimático. O nível de significância aceitável para os resultados foi p <0,05.

**Resultados:**

A dieta hiperglicídica promoveu o desenvolvimento de síndrome metabólica. Tal fato culminou no remodelamento hipertrófico do músculo liso vascular e tecido adiposo perivascular. Além disso, houve aumentos das citocinas TNF-α e IL-6 circulantes e no tecido adiposo perivascular. O tratamento com atorvastatina reduziu significativamente os danos metabólicos, o remodelamento vascular e os níveis de citocinas.

**Conclusão:**

Atorvastatina ameniza danos metabólicos associados à síndrome metabólica induzida por dieta hiperglicídica, além de atenuar o remodelamento vascular, sendo esses efeitos associados à redução de citocinas pró-inflamatórias.

## Introdução

As doenças cardiovasculares representam grandes riscos para a qualidade de vida da população no mundo. No Brasil, aproximadamente 350 mil indivíduos morrem por doenças cardiovasculares anualmente.^1 , 2^ Dentre as diversas causas predisponentes para riscos cardiovasculares, destaca-se a síndrome metabólica (SM), caracterizada por um conjunto de comorbidades que envolvem aumento da circunferência abdominal (≥89 cm para mulheres e ≥102 cm para homens), aumento nos níveis de triglicerídeos (≥150 mg/dL), redução nos níveis de colesterol HDL (≤50 mg/dL para mulheres e ≤40 mg/dL para homens), aumento nos níveis pressóricos (≥130/85 mmHg) e aumento na glicemia de jejum (≥100 mg/dL). Dentre estas, a presença de pelo menos três comorbidades é elementar para o diagnóstico de SM.^3 , 4^

Durante a SM, observam-se alterações estruturais e funcionais dos componentes do sistema vascular.^5 - 7^ Em indivíduos com SM, há disfunção endotelial, além de aumento na migração e proliferação de células musculares lisas. Observa-se também a expansão do tecido adiposo perivascular (PVAT), evidenciada pelo aumento morfológico dos adipócitos, bem como a substituição do tecido adiposo marrom pelo tecido adiposo branco, resultando na diminuição da liberação de fatores relaxantes derivados do PVAT e consequente perda da sua capacidade de ação anticontrátil.^8 - 11^

A hipertrofia e a substituição dos adipócitos no PVAT são capazes de favorecer a produção e o acúmulo de citocinas pró-inflamatórias.^12^ Roedores submetidos à dieta hipercalórica e que desenvolveram SM mostraram aumento e acúmulo do PVAT nas artérias aorta e carótida. Tal fato promoveu a liberação de quimiocinas e recrutamento de monócitos e células T para o PVAT.^13^ Neste ambiente inflamatório, também é possível observar o aumento na produção de interleucina-6 (IL-6) e o infiltrado de macrófagos secretores do fator de necrose tumoral alfa (TNF-α) no PVAT.^14^ Este conjunto desencadeia a inflamação crônica do sistema vascular, prejudicando a estrutura e a função dos seus constituintes.

A inflamação dos vasos sanguíneos, de fato, contribui para o remodelamento vascular, aumento da resistência periférica e desordens circulatórias.^7 , 15 , 16^ Neste contexto, o remodelamento vascular é um processo crônico adaptativo, caracterizado por alterações na estrutura dos vasos sanguíneos, derivadas de citocinas pró-inflamatórias, além de interações entre fatores de crescimento, estímulos hemodinâmicos e espécies reativas de oxigênio. Compreende modificações no crescimento, morte e migração celular, e na síntese e degradação da matriz extracelular.^17 - 19^

Um fator importante a ser considerado nas alterações vasculares encontradas durante a SM é a dislipidemia, uma condição caracterizada pelo aumento de colesterol e triglicerídeos na circulação e redução dos níveis de HDL.^20^ Numerosos estudos estabeleceram que a dislipidemia leva a uma resposta inflamatória na vasculatura, refletida pela ativação das células endoteliais, recrutamento de leucócitos e produção de citocinas pró-inflamatórias.^21 , 22^ Camundongos hipercolesterolêmicos alimentados com dieta hiperlipídica mostram remodelamento da camada média da artéria femoral, associado ao recrutamento de macrófagos.^23^ Neste contexto, há correlação positiva entre os níveis de colesterol circulantes, inflamação crônica e remodelamento vascular.

Atorvastatina, inibidora da β-hidroxi-β-metil-glutaril coenzima A redutase, se apresenta como uma das estatinas mais eficazes no tratamento das dislipidemias, reduzindo a produção de colesterol LDL e aumentando a produção de colesterol HDL.^24 , 25^ Não obstante, nas últimas décadas, crescentes evidências, sejam experimentais ou clínicas, se acumularam para apoiar a ideia de que a atorvastatina exerce efeitos cardiovasculares benéficos, independentemente de seus efeitos primários.^26^ Em um modelo experimental de SM, a atorvastatina foi capaz de melhorar a reatividade e reduzir o remodelamento estrutural de artérias de resistência; ambos os efeitos foram associados à redução da inflamação e do estresse oxidativo.^27^ Além disso, a atorvastatina é capaz de inibir a secreção de metaloproteinase de matriz-9 em células vasculares e suprimir a expressão do fator de transformação do crescimento beta, diminuindo a fibrose vascular.^28 - 30^ Esses efeitos *in vitro* da atorvastatina também estão associados à sua capacidade anti-inflamatória.^31^ No contexto clínico, a atorvastatina reduz processos ateroscleróticos^32^ e quadros clínicos de síndrome coronária aguda.^33^ De fato, dentre os diversos efeitos pleiotrópicos da atorvastatina, é robusta a sua capacidade vasoprotetora. Ainda assim, não há evidências suficientes que atestam sobre os efeitos e os possíveis mecanismos envolvidos nas ações da atorvastatina, sob a perspectiva do músculo liso vascular e, de forma inédita, sob o PVAT como tecidos inflamatórios em condições de SM. Assim, neste estudo, hipotetizamos que, durante a SM, ocorre remodelamento vascular a níveis de músculo liso vascular e PVAT, ocasionado pelo aumento da inflamação vascular. Além disso, o tratamento com atorvastatina pode ser capaz de reduzir a inflamação vascular e, consequentemente, reverter os prejuízos associados à SM.

## Métodos

### Animais e delineamento experimental

Todos os procedimentos experimentais foram analisados pela Comissão de Ética no Uso de Animais da Universidade Federal de Jataí (Protocolo 02/2019). Camundongos Swiss machos, de 3 semanas de idade, foram adquiridos do Biotério Central da Universidade Federal de Goiás – Regional Goiânia – e acondicionados no Biotério de Experimentação Animal da Universidade Federal de Jataí, com temperatura controlada de 22 ± 2ºC, umidade de 50 ± 5% e ciclos claro-escuro de 12 horas, com livre acesso a água e alimento. Os camundongos foram mantidos durante todo o período experimental em caixas de polipropileno (comprimento [30] × largura [20] × altura [13] cm), na proporção de 3 camundongos por caixa.

Após uma semana de aclimatização, os camundongos receberam dieta controle ou dieta hiperglicídica por 18 semanas. Após 14 semanas de dietas, os camundongos foram tratados (diariamente/turno vespertino) por gavagem com veículo (solução salina) ou atorvastatina [(20mg/kg), Sigma-Aldrich, #PZ0001, Alemanha] durante 4 semanas.^34^ A dieta controle constitui-se em 22% de proteína, 70% de carboidrato e 8% de gordura, enquanto a dieta hiperglicídica constitui-se em 10% de proteína, 80% de carboidrato e 10% de gordura. A dieta hiperglicídica foi formada por 33% de dieta controle (Nuvilab® CR1, Nuvital, Brasil), 33% de leite condensado e 7% de sacarose por peso da dieta controle, sendo o restante água.^6^ O conteúdo energético foi de 12,16 kJ/g para a dieta controle e 13,35 kJ/g para a dieta hiperglicídica. Os camundongos foram eutanasiados por excesso de anestésicos (cetamina e xilazina, 140 mg/kg e 12 mg/kg, respectivamente, via intraperitoneal).

### Perfil nutricional e murinométrico do modelo experimental

O perfil nutricional foi determinado pelo consumo de alimentos e consequente ingestão calórica, além da eficiência alimentar. A ingestão calórica (por camundongo) foi calculada a partir da ingestão semanal de alimentos, multiplicada pelo valor energético da dieta (g × kcal). Com a finalidade de analisar a capacidade de o camundongo converter a energia consumida em massa corporal, a eficiência alimentar foi calculada, dividindo-se o ganho total de massa corporal (g) pela energia total ingerida (kcal), em porcentagem. O perfil murinométrico foi determinado pela análise da massa e gordura corporal. A massa corporal dos camundongos foi aferida semanalmente, utilizando balança analítica de precisão, e a obesidade foi caracterizada, ao final das dietas e tratamentos, pelo índice de adiposidade {[gordura corporal (g)/peso corporal final (g)]×100}, sendo a gordura corporal calculada pela soma das gorduras epididimal, retroperitoneal e visceral.^35^

### Obtenção de soro e PVAT

Os camundongos foram submetidos à privação alimentar por 8 horas. Após eutanásia, amostras de sangue foram coletadas por punção cardíaca e transferidas para tubos secos. O sangue foi centrifugado a 2.500 rpm por 15 minutos para separação do soro, utilizado nas dosagens bioquímicas e citocinas. O PVAT foi retirado mecanicamente dos segmentos de aorta torácica, congelado em nitrogênio líquido, pulverizado e homogeneizado em tampão fosfato salina (PBS) gelado. O extrato tecidual foi centrifugado a 13.000 rpm por 20 minutos para separação do material insolúvel. O sobrenadante foi coletado para as dosagens de citocinas.

### Avaliação do perfil lipídico e glicêmico

Foram determinados os níveis séricos de colesterol total, triglicerídeos e HDL (lipoproteínas de alta densidade), por métodos enzimáticos ( *kits* Labtest, Brasil), em amostras de soros provenientes da centrifugação do sangue total. A partir da concentração sérica de triglicerídeos, foram calculadas as concentrações de VLDL (lipoproteínas de muita baixa densidade), utilizando-se o seguinte cálculo: VLDL (mg/dL) = triglicerídeos (mg/dL)/5. A partir da concentração de HDL e VLDL, foi calculada a concentração de LDL (lipoproteínas de baixa densidade), utilizando o cálculo: LDL (mg/dL) = colesterol total – HDL – VLDL.^36^ A glicemia foi determinada após o tratamento com dietas. Uma gota de sangue foi colocada em fitas individuais específicas para leitura em glicosímetro (Accu-Check Active, Alemanha).

### Teste oral de tolerância à glicose (TOTG)

Um dia anterior ao final da 18ª semana de dietas e tratamentos, após 8 horas de jejum, a glicemia de cada camundongo foi verificada por glicosímetro (Accu-Check Active, Alemanha), caracterizando a glicemia do tempo zero. Em seguida, glicose na dose de 2 g/kg foi administrada aos camundongos por gavagem. A partir desse momento, o cronômetro foi acionado e novas determinações da glicemia foram feitas nos tempos de 30, 60, 90 e 120 minutos.^37^

### Avaliação morfoestrutural do músculo liso vascular e do PVAT

Aortas da região torácica foram removidas e então fixadas por 24 horas em paraformaldeído a 4%. As etapas seguintes envolveram a desidratação em concentrações crescentes de álcool etílico (70%, 80%, 90% e 100%) por 120 minutos, com posterior diafanização em álcool etílico absoluto cada (1:1) 1, 2 e 3 por 120 minutos e em xilol 1, 2 e 3 por 30 minutos, com posterior inclusão em parafina. As aortas foram incluídas em parafina e cortadas em micrótomo na espessura de 4,5 µm, para posterior coloração.

Para a coloração de hematoxilina e eosina, os cortes em lâminas foram submetidos ao processo de desparafinização, sendo mantidos em estufa a 65ºC, posterior imersão em xilol 1 e 2 por 20 minutos. Em seguida, foram imergidos em soluções decrescentes (100%, 90%, 70% e 50%) de álcool etílico durante 5 minutos e imersos em água destilada por 10 minutos. Seguida a hidratação, as lâminas foram imersas em corante hematoxilina por 6 minutos e, logo após, lavadas em água corrente por 10 minutos. A seguir, foram imersas em corante eosina por 6 minutos. Os cortes corados foram novamente desidratados e diafanizados, sendo montados com meio de montagem Permount^®^ (Fisher Scientific, EUA) e lamínulas.

Imagens coloridas foram obtidas por câmera digital acoplada à microscópio óptico e analisadas pelo programa ImageJ (Instituto Nacional de Saúde, EUA). Para análise das imagens, foi delimitado o lúmen, a túnica média e adventícia com o objetivo de calcular as seguintes variáveis morfométricas: área de secção transversal (AST) da túnica média e espessura da túnica média. O volume dos adipócitos (v) foi calculado a partir da fórmula v = πd^3^ /6, em que (d) representa o diâmetro dos adipócitos. A massa do adipócito se deu pelo volume multiplicado pela densidade (0,92 g/cm^3^ ). O número de adipócitos foi determinado dividindo-se a massa do PVAT pela massa média de adipócitos.^38^

### Determinação dos níveis de citocinas inflamatórias

Os níveis de TNF-α e IL-6 no soro e no PVAT foram feitos pelo método imunoenzimático (ELISA) utilizando *kits* DuoSet ELISA Development Systems (R&D Systems, EUA) de acordo com as informações do fabricante. Placas de microtitulação (96 poços) foram recobertas com 50 μL/poço dos anticorpos específicos anti-TNF-α e anti-IL-6, nas concentrações descritas pelo fabricante, diluídos em PBS e incubados *overnight* a 4ºC. As placas foram lavadas com PBS/Tween-20 (0,05%), e as ligações não específicas foram bloqueadas com 100 μL de PBS contendo albumina do soro bovino 1% durante 2 horas em temperatura ambiente. Posteriormente, as amostras foram adicionadas, seguindo nova incubação por 2 horas em temperatura ambiente. Após esse período, as placas foram lavadas e foram adicionados 50 μL dos anticorpos biotinilados específicos para cada citocina. Após 2 horas, as placas foram lavadas e o conjugado estreptavidina-peroxidase, na diluição de 1:40, foi adicionado a cada poço. As placas foram incubadas por 1 hora em temperatura ambiente. Posteriormente, as placas foram lavadas e adicionou-se a dosagem de 100 μL do substrato tetrametilbenzidina. A densidade ótica foi medida a 630 nm no espectrofotômetro SpectraMAX 190 Microplate Reader (Molecular Devices, EUA). Os níveis de citocinas contidas nas amostras foram calculados a partir de uma curva padrão com 11 pontos obtidos por diluição seriada. Os resultados foram expressos em pg/mL para amostras de soro, e pg/mg de proteína para amostras de PVAT.

### Análise estatística

Os resultados foram expressos como média ± desvio padrão (DP). Os dados seguiram distribuição normal de acordo com o teste de Kolmogorov-Smirnov. Os resultados foram analisados pelo teste de análise de variância de duas vias (Two-Way ANOVA), seguido do pós-teste Tukey. O nível de significância mínima aceitável foi p <0,05. Por conveniência, o número de camundongos para cada grupo experimental foi igual a 6. O programa Prisma, versão 8.0 (GraphPad Software Inc., EUA) foi utilizado para analisar esses parâmetros, bem como para a construção dos gráficos.

## Resultados

### Perfil nutricional e murinométrico

**Figura 1:**
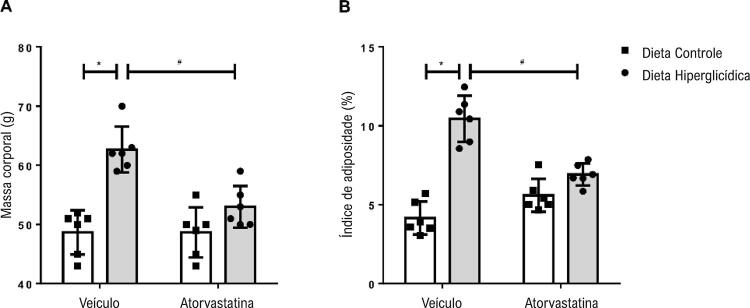
Atorvastatina atenua a obesidade associada à síndrome metabólica induzida por dieta hiperglicídica. As figuras mostram valores de massa corporal (A) e índice de adiposidade (B) de camundongos mantidos por 18 semanas em dieta controle ou dieta hiperglicídica, tratados com veículo ou atorvastatina (20 mg/kg por 4 semanas). Os resultados representam a média ± DP. *, p <0,05 vs. dieta controle_veículo; #, p <0,05 vs. dieta hiperglicídica_veículo (n = 6 para cada grupo experimental).

A ingestão alimentar e a consequente ingestão calórica foram maiores nos camundongos mantidos com dieta hiperglicídica, bem como a eficiência alimentar; no entanto, este último parâmetro foi reduzido após o tratamento com atorvastatina. Os parâmetros murinométricos, como ganho de massa, depósitos de gorduras epididimal, retroperitoneal e visceral foram maiores nos camundongos mantidos em dieta hiperglicídica quando comparados aos camundongos mantidos em dieta controle. O tratamento com atorvastatina promoveu redução de tais parâmetros ( Tabela 1 ). Os camundongos mantidos em dieta hiperglicídica apresentaram maior aumento de massa corporal quando comparados aos camundongos mantidos em dieta controle. Além disso, camundongos mantidos em dieta hiperglicídica e tratados com atorvastatina apresentaram redução de massa corporal ( Figura 1A ). O índice de adiposidade, parâmetro para determinar a obesidade, foi maior nos camundongos mantidos em dieta hiperglicídica quando comparados aos camundongos mantidos em dieta controle. O tratamento com atorvastatina promoveu redução no índice de adiposidade dos camundongos mantidos em dieta hiperglicídica ( Figura 1B ).

**Tabela 1:** – Comportamento alimentar e avaliação ponderal dos depósitos de gordura corporal

	Dieta C + Veículo	Dieta C + Atorvastatina	Dieta H + Veículo	Dieta H + Atorvastatina
Ingestão alimentar (g/semana)	22,6 ± 0,3	21,3 ± 0,4	31,7 ± 0,2	31,1 ± 0,5
Ingestão calórica (kcal/semana)	274,8 ± 3,7	259,1 ± 4,9	423,1 ± 2,67*	415,2 ± 6,7
Eficiência alimentar (%)	0,51 ± 0,01	0,53 ± 0,01	0,64 ± 0,02*	0,47 ± 0,03^#^
Ganho de massa (g)	25,0 ± 2,31	24,5 ± 2,25	37,3 ± 1,52*	27,9 ± 1,22^#^
Gordura epididimal (g)	0,97 ± 0,12	1,13 ± 0,11	2,68 ± 0,24*	1,31 ± 0,09^#^
Gordura retroperitoneal (g)	0,32 ± 0,06	0,61 ± 0,19	1,88 ± 0,12*	0,93 ± 0,19^#^
Gordura visceral (g)	0,75 ± 0,15	0,94 ± 0,13	1,67 ± 0,11*	1,01 ± 0,17^#^

Dados expressos como média ± DP. *, p <0,05 vs. dieta C + veículo; ^#^, p <0,05 vs. dieta H + veículo (n = 6 para cada grupo experimental). Dieta C: dieta controle, dieta H: dieta hiperglicídica.

### Perfil lipídico

**Figura 2:**
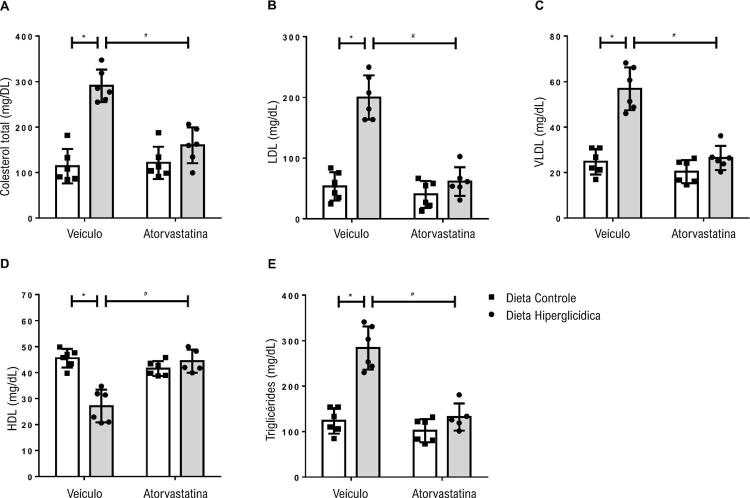
– Atorvastatina atenua a dislipidemia associada à síndrome metabólica induzida por dieta hiperglicídica. As figuras mostram níveis de colesterol total (A), LDL (B), VLDL (C), HDL (D) e níveis de triglicerídeos (E) de camundongos mantidos por 18 semanas em dieta controle ou dieta hiperglicídica, tratados com veículo ou atorvastatina (20 mg/kg por 4 semanas). Os resultados representam a média ± DP. *, p <0,05 vs. dieta controle_veículo; #, p <0,05 vs. dieta hiperglicídica_veículo (n = 6 para cada grupo experimental).

Os camundongos mantidos em dieta hiperglicídica apresentaram dislipidemia quando comparados aos camundongos que receberam dieta controle. A dislipidemia pode ser observada a partir do aumento nos níveis de colesterol total ( Figura 2A ), em que as frações LDL ( Figura 2B ) e VLDL ( Figura 2C ) se encontraram aumentadas e a fração HDL ( Figura 2D ), diminuída. Houve também aumento nos níveis de triglicerídeos dos camundongos que receberam dieta hiperglicídica em comparação aos que foram mantidos em dieta controle ( Figura 2E ). Os camundongos mantidos em dieta hiperglicídica e que foram tratados com atorvastatina mostraram redução de tais parâmetros lipídicos.

### Perfil glicêmico

**Figura 3:**
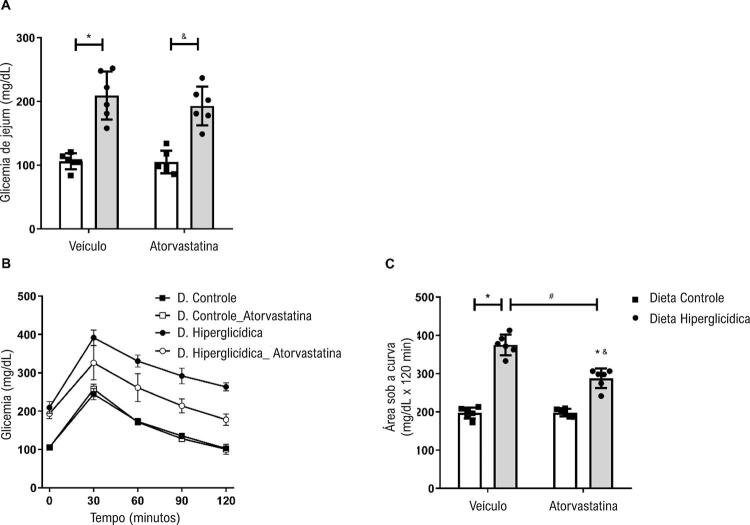
– Atorvastatina atenua a intolerância à glicose associada à síndrome metabólica induzida por dieta hiperglicídica. As figuras mostram níveis de glicemia em jejum (A), curvas glicêmicas (B) e área sob a curva (C), obtidas no teste oral de tolerância à glicose de camundongos mantidos por 18 semanas em dieta controle ou dieta hiperglicídica, tratados com veículo ou atorvastatina (20 mg/kg por 4 semanas). Os resultados representam a média ± DP. *, p <0,05 vs. dieta controle_veículo; &, p <0,05 vs. dieta controle_atorvastatina; #, p <0,05 vs. dieta hiperglicídica_veículo (n = 6 para cada grupo experimental).

A glicemia de jejum dos camundongos mantidos em dieta hiperglicídica estava aumentada quando comparada à glicemia de jejum dos camundongos mantidos em dieta controle. Os camundongos que receberam dieta hiperglicídica e tratamento com atorvastatina não mostraram redução na concentração glicêmica ( Figura 3A ). No TOTG ( Figura 3B ), camundongos que receberam dieta hiperglicídica apresentaram aumento na concentração glicêmica quando comparados aos camundongos que receberam dieta controle, sendo que, mesmo com o passar do tempo, os níveis de glicose não retornaram aos níveis basais, como observado nos camundongos que receberam dieta controle. Além disso, camundongos que receberam dieta hiperglicídica e tratados com atorvastatina apresentaram, com o passar do tempo, menor glicemia. A área sob a curva reflete os efeitos citados ( Figura 3C ).

### Morfologia muscular lisa

**Figura 4:**
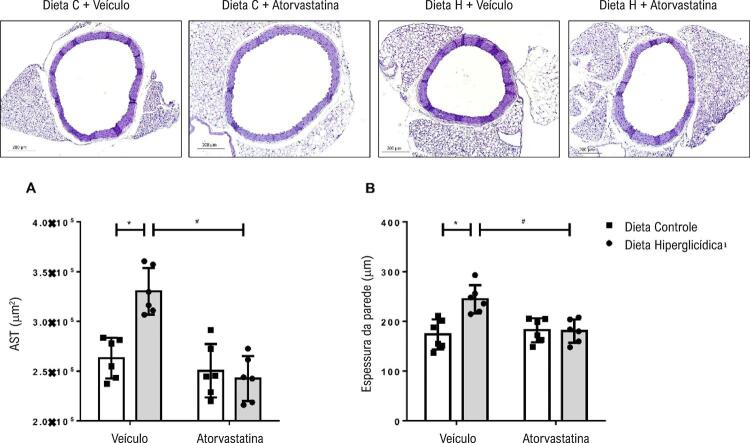
– Atorvastatina atenua o remodelamento do músculo liso vascular associado à síndrome metabólica induzida por dieta hiperglicídica. As figuras mostram imagens representativas, tamanho da AST (A) e espessura da parede do vaso (B) de camundongos mantidos por 18 semanas em dieta controle ou dieta hiperglicídica, tratados com veículo ou atorvastatina (20 mg/kg por 4 semanas). Os resultados representam a média ± DP. *, p <0,05 vs. dieta controle_veículo; #, p <0,05 vs. dieta hiperglicídica_veículo (n = 6 para cada grupo experimental). Dieta C: dieta controle, Dieta H: dieta hiperglicídica.

Camundongos mantidos em dieta hiperglicídica apresentaram aumento na AST vascular quando comparados aos camundongos mantidos em dieta controle. Camundongos que receberam dieta hiperglicídica e foram tratados com atorvastatina mostraram redução na AST ( Figura 4A ). De modo similar, camundongos que receberam dieta hiperglicídica apresentaram aumento na espessura da camada média do vaso quando comparados aos camundongos que receberam dieta controle. O tratamento com atorvastatina foi capaz de reduzir este aumento na espessura da camada média ( Figura 4B ).

### Morfologia do tecido adiposo perivascular

**Figura 5:**
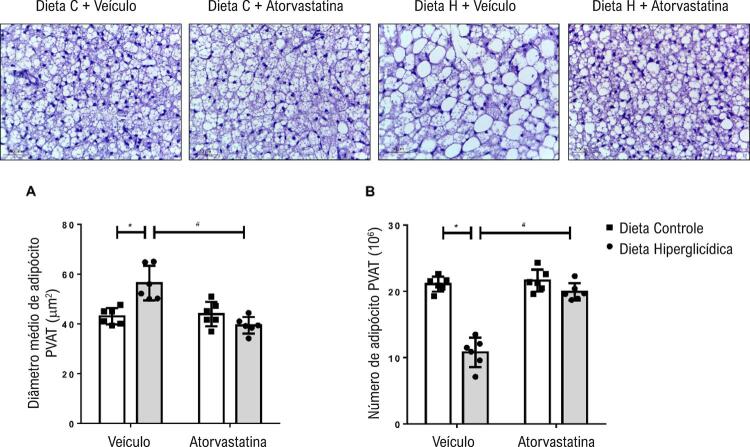
– Atorvastatina atenua o remodelamento do PVAT associado à síndrome metabólica induzida por dieta hiperglicídica. As figuras mostram imagens representativas (A) e o tamanho de adipócitos PVAT (B) de camundongos mantidos por 18 semanas em dieta controle ou dieta hiperglicídica, tratados com veículo ou atorvastatina (20 mg/kg por 4 semanas). Os resultados representam a média ± DP. *, p <0,05 vs. dieta controle_veículo; #, p <0,05 vs. dieta hiperglicídica_veículo (n = 6 para cada grupo experimental). Dieta C: dieta controle, Dieta H: dieta hiperglicídica.

Para verificar se houve alterações na morfologia do PVAT, foi avaliada a túnica adventícia a partir da medida e do número dos adipócitos presentes na mesma, sendo possível observar que os camundongos que receberam dieta hiperglicídica apresentaram adipócitos maiores ( Figura 5A ) e em menor número ( Figura 5B ) quando comparados com os camundongos que receberam dieta controle. O grupo que recebeu dieta hiperglicídica e tratamento com atorvastatina obteve redução no tamanho e aumento no número dos adipócitos.

### Perfil inflamatório

**Figura 6:**
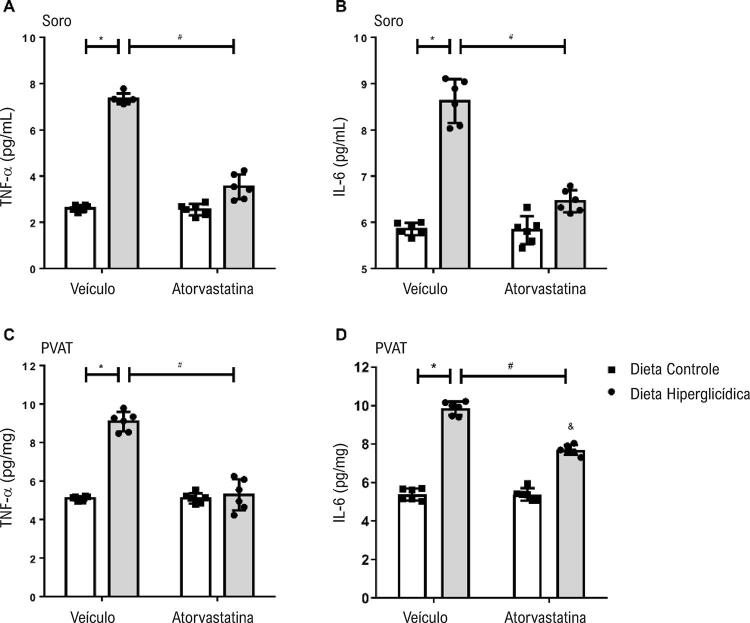
– Atorvastatina atenua os aumentos nas concentrações de citocinas pró-inflamatórias associados à síndrome metabólica induzida por dieta hiperglicídica. As figuras mostram níveis de TNF-α (A e C) e IL-6 (B e D) no soro e no PVAT, respectivamente, de camundongos mantidos por 18 semanas em dieta controle ou dieta hiperglicídica, tratados com veículo ou atorvastatina (20 mg/kg por 4 semanas). Os resultados representam a média ± DP. *, p <0,05 vs. dieta controle_veículo; &, p <0,05 vs. dieta controle_atorvastatina; #, p <0,05 vs. dieta hiperglicídica_veículo (n = 6 para cada grupo experimental).

Foram determinadas as concentrações de citocinas pró-inflamatórias TNF-α ( Figuras 6A e 6C ) e IL-6 ( Figuras 6B e 6D ) no soro e no PVAT. Os camundongos que receberam dieta hiperglicídica apresentaram maiores concentrações de TNF-α e IL-6, tanto no soro quanto no PVAT, quando comparados aos camundongos que receberam dieta controle. O tratamento com atorvastatina reduziu os níveis de TNF-α e IL-6, tanto no soro quanto no PVAT, de camundongos mantidos em dieta hiperglicídica.

## Discussão

A dieta hiperglicídica resultou em mudanças murinométricas e metabólicas em camundongos. Silva et al.^39^ submeteu um modelo experimental à dieta semelhante, verificando aumentos em idênticos parâmetros utilizados neste estudo.^39^ Neste contexto, Leopoldo et al.,^40^ utilizando modelos experimentais, definiu as categorias de adiposidade após submissão à dieta hiperglicídica, tais quais variam entre: normal – índice de adiposidade (%) 4,17 a 4,55; excesso de peso – índice de adiposidade (%) 5,69 a 6,23; e obesidade – índice de adiposidade (%) 7,53 a 10,02.^40^ Nossos resultados demostram índice de adiposidade de aproximadamente 10% para os camundongos mantidos em dieta hiperglicídica, o que classifica o modelo experimental como obeso.

A glicose é um carboidrato que atua como uma das principais fontes de energia para o organismo e, em excesso, através de vias metabólicas, é armazenada em forma de glicogênio. Se a capacidade de armazenamento da ingesta de carboidratos excede a glicogênese, o excedente é convertido em triacilgliceróis e é incorporado a colesteróis provenientes da dieta e a apolipoproteínas, responsáveis pelo transporte de triglicerídeos até os tecidos de armazenamento, como, por exemplo, o tecido adiposo.^41^ Dessa forma, o excesso de carboidratos ofertados na dieta dos camundongos corrobora para o aumento da massa corporal e do índice de adiposidade.

Os camundongos mantidos em dieta hiperglicídica apresentaram dislipidemia e aumento da glicose sérica em jejum. Mullugeta et al.,^42^ demostrou, em portadores de diabetes melito do tipo 2, que a hiperglicemia e a resistência à insulina estão associados à dislipidemia, ou seja, hiperglicemia promove prejuízos no metabolismo lipídico. Além disso, demostrou-se que indivíduos hiperglicêmicos apresentam valores séricos de triglicerídeos, colesterol total e frações aumentados quando comparados aos indivíduos normoglicêmicos.^42 , 43^

O tratamento com atorvastatina se mostrou eficiente para o controle dos níveis de colesterol total e frações. Além disso, a atorvastatina reduziu os níveis de triglicerídeos, a massa corporal e o índice de adiposidade. Silva et al.^44^ também demostrou a eficácia da atorvastatina em doses diárias de 10 mg ou 20 mg sobre a correção do perfil lipídico de indivíduos, em que, além da terapêutica sobre o colesterol, os triglicerídeos também foram reduzidos.^44^ Sabe-se que VLDL e LDL são lipoproteínas constituídas por quantidades significativas de triacilgliceróis; sendo assim, propomos que, com a redução dos níveis de VLDL e LDL, por meio da ação da atorvastatina, é observada diminuição de triglicerídeos. Parhofer et al.^45^ descreve o efeito redutor de triglicerídeos principalmente pela a inibição da síntese de colesterol, e também salientam que VLDL e LDL têm o mesmo mecanismo de remoção. Assim, ao reduzir a quantidade de LDL na circulação sanguínea, pode-se também diminuir significantemente os níveis de VLDL.^45^

A dieta hiperglicídica, de acordo com o TOTG, induziu nos camundongos intolerância à glicose. Sabe-se que a exposição crônica do organismo à hiperglicemia acaba afetando a atividade insulínica no que diz respeito a síntese, secreção e ação deste hormônio, o que, de fato, é observado no diabetes melito tipo 2.^6^ Sendo assim, é possível sugerir que os resultados levantados no perfil glicêmico de camundongos mantidos em dieta hiperglicídica corroboram para o desenvolvimento de diabetes melito tipo 2. Kissebah et al.,^46^ demonstrou que o aumento da gordura visceral está associado à intolerância à glicose e à resistência à insulina; Taylor et al.,^47^ demonstrou que a gordura visceral está relacionada com o desenvolvimento de diabetes melito.^46 , 47^ Estudos descrevem que o aumento da gordura visceral predispõe a produção e secreção de citocinas, tais como IL-6 e TNF-α, e adipocinas como resistina e adiponectina, que estão associadas ao desenvolvimento de resistência à insulina e disfunções no metabolismo e no controle da glicose, por interferência na sinalização intracelular na insulina.^48 , 49^ O aumento da gordura visceral, bem como o aumento de TNF-α e IL-6, justificam o desequilíbrio no perfil glicêmico.

A terapia com atorvastatina foi capaz de atenuar a intolerância à glicose nos camundongos mantidos em dieta hiperglicídica. Huptas, et al.^50^ obteve resultados promissores em relação ao tratamento de indivíduos portadores de SM com atorvastatina, ele demostrou melhoras no metabolismo da glicose mediante a redução da intolerância à glicose e da resistência à insulina. Suzuki et al.,^51^ utilizando modelos experimentais de intolerância à glicose e de resistência à insulina, realizou TOTG e teste de tolerância à insulina (TTI) com ou sem o tratamento com atorvastatina, e demostrou que o grupo tratado tem TOTG e TTI reduzidos em comparação ao grupo que recebeu veículo.^50 , 51^

Não se sabe ao certo por quais mecanismos a atorvastatina melhora os parâmetros relacionados ao metabolismo da glicose. Tal melhora pode estar relacionada à diminuição do processo de gliconeogênese.^51^ No entanto, é possível sugerir que tal redução da intolerância à glicose pode estar relacionada com a redução dos depósitos de gordura visceral; além disso, pode estar relacionada à redução da produção de citocinas (TNF-α e IL-6), que são potenciais causas de disfunções metabólicas.

De acordo com Mulvany (1999), o remodelamento vascular pode ser classificado a partir de medidas da AST e espessura da parede. O aumento da AST e da espessura da parede é característico de remodelamento do tipo hipertrófico.^52^ Uma das causas do remodelamento vascular é a inflamação mediada por aumento de citocinas pró-inflamatórias. Estudos relacionados à SM mostram que a hipertrofia do PVAT resulta na migração, ativação de células imunes e aumento da secreção dessas citocinas, levando a um processo inflamatório crônico de baixa intensidade.^53 - 55^

As mudanças na estrutura e na composição do PVAT refletem-se na túnica média do vaso, pois, fisiologicamente, o PVAT é constituído de adipócitos que agem como reguladores da proliferação de células da musculatura lisa vascular (CMLV). A mudança por adipócitos não especializados culmina em aumento da proliferação de CMLV e hipertrofia da musculatura lisa. Esta hipertrofia apresenta papel significativo em doenças vasculares como, por exemplo, reestenose e hipertensão arterial.^56^

TNF-α e IL-6 se encontram em concentrações aumentadas durante a inflamação crônica presente na obesidade, na resistência à insulina e na SM. Além disso, há clara relação do aumento dessas citocinas em mudanças fenotípicas do PVAT.^57 , 58^ Citocinas pró-inflamatórias promovem o remodelamento e a disfunção vascular, uma vez que concentrações aumentadas de TNF-α promovem a hiperplasia neointimal e a disfunção endotelial. De forma semelhante, a IL-6, quando em concentrações aumentadas, pode induzir a infiltração de macrófagos no PVAT, contribuindo para o desenvolvimento de aneurisma e remodelamento vascular associado à inflamação do PVAT.^59 , 60^

A partir de uma dieta hiperlipídica, foi induzida em coelhos hipercolesterolemia para levantamento das alterações que a condição poderia desenvolver nas artérias pulmonares dos animais. As artérias apresentaram aumento da proliferação de CMLV, bem como hipertrofia da túnica média e hiperplasia da túnica íntima, além disso, no tecido pulmonar, havia grande infiltrado de células inflamatórias como, por exemplo, macrófagos; houve aumento de IL-6 no soro desses animais. Os coelhos foram tratados com atorvastatina, evidenciando-se a reversão da condição hipercolesterolêmica, do remodelamento vascular e dos processos inflamatórios no tecido pulmonar.^61^ Similarmente, atorvastatina inibe o remodelamento vascular induzido por aldosterona, via redução de citocinas pró-inflamatórias.^31^

Como limitação desse estudo, não é possível estabelecer uma relação casuística direta entre o tratamento farmacológico com atorvastatina, as amenidades no perfil metabólico e o remodelamento vascular. A partir do delineamento experimental utilizado nesse estudo, mostramos que a atorvastatina reduz a adiposidade e melhora os perfis lipídicos, glicêmicos e inflamatórios. Na literatura, é clássico que tais fatos são benéficos para a estrutura e a função vascular. Sendo assim, não temos a clareza absoluta de que o remodelamento vascular atenuado se deu por ações diretas da atorvastatina sobre a vasculatura, ou por meio de efeitos adjacentes ao metabolismo. Ressaltamos que, em organismos multicomplexos, mecanismos isolados não justificam a gênese de doenças, sendo que, nesse contexto, há sempre uma vasta interação entre mecanismos e sistemas para tal. Portanto, independentemente da ação direta ou pleiotrópica da atorvastatina, a terapia final se mostra promissora para o contexto deste estudo.

## Conclusão

Em resumo, este estudo mostra que o tratamento com atorvastatina atenua os danos metabólicos associados à síndrome metabólica induzida por dieta hiperglicídica, além de atenuar o remodelamento do músculo liso vascular e do PVAT, sendo esses efeitos associados à redução de citocinas pró-inflamatórias TNF-α e IL-6.
