# Supplementary material for: Atorvastatina Atenua o Remodelamento Vascular em Camundongos com Síndrome Metabólica
Source: Arq Bras Cardiol. 2021 Jun 10;117(4):737–47. [Article in Portuguese] doi: 10.36660/abc.20200322 (PMC8528348; doi:10.36660/abc.20200322)
Supplement: Translation

# Translation: Atorvastatin Attenuates Vascular Remodeling in Mice with Metabolic Syndrome

**Authors:** Karine Ferreira da Silva Carvalho, Amanda Araújo Marques Ferreira, Nayara Carvalho Barbosa, Juliano Vilela Alves, Rafael Menezes da Costa

**Affiliations:** 1 Brazil Universidade Federal de Jataí - Unidade Acadêmica de Ciências da Saúde, Jataí, GO - Brazil; 2 Brazil Faculdade de Medicina de Ribeirão Preto, Universidade de São Paulo - Departamento de Farmacologia, Ribeirão Preto, SP - Brazil

**Keywords:** Dyslipidemias, Dietary Carbohydrates, Metabolic Syndrome, Vascular Remodeling, Atorvastatin, Inflammation, Mice

## Abstract

**Background:**

Metabolic syndrome is characterized by an array of comorbidities. During this syndrome, structural changes are observed in the cardiovascular system, especially vascular remodeling. One of the predisposing causes for these changes is chronic inflammation resulting from changes in the structure and composition of perivascular adipose tissue. Atorvastatin is effective in the treatment of dyslipidemias. However, its pleiotropic effects have not been completely understood. We hypothesize that metabolic syndrome may lead to vascular remodeling and that atorvastatin therapy may be able to attenuate this condition.

**Objectives:**

To assess the effects of atorvastatin therapy on vascular remodeling in an experimental model of metabolic syndrome.

**Methods:**

Swiss mice received a control diet or a hyperglicemic diet for 18 weeks. After 14 weeks of diet, mice were treated with vehicle or atorvastatin (20mg/kg) during for 4 weeks. Nutritional and metabolic profiles were assessed by biochemical tests; moreover, a histological assessment of aorta structure was conducted, and cytokine levels were determined by the immunoenzyme assay. The acceptable level of significance for the results was set at p<0.05.

**Results:**

Hyperglicemic diet promoted the development of metabolic syndrome. It indeed culminated in hypertrophic remodeling of vascular smooth muscle and perivascular adipose tissue. Furthermore, there were increases in the levels of circulating TNF-α and IL-6 and in the perivascular adipose tissue. Atorvastatin therapy significantly reduced metabolic damages, vascular remodeling, and cytokine levels.

**Conclusion:**

Atorvastatin attenuate metabolic damages associated with metabolic syndrome induced by hyperglycemic diet, in addition to attenuating vascular remodeling; both effects are associated with reduced levels of pro-inflammatory cytokines.

## Introduction

Cardiovascular diseases pose great risks for the quality of life of the world population. In Brazil, nearly 350 thousand individuals die from cardiovascular diseases every year.^1 , 2^ One of the most important predisposing causes for cardiovascular risks is metabolic syndrome (MS), characterized by an array of comorbidities involving increased abdominal circumference (≥ 89 cm for women and ≥ 102 cm for men), increased levels of triglycerides (≥ 150 mg/dL), reduced levels of HDL cholesterol (≤ 50 mg/dL for women and ≤ 40 mg/dL for men), increased levels of blood pressure (≥ 130/85 mmHg), and increased levels of fasting glucose (≥ 100 mg/dL). The presence of at least three of these comorbidities is pivotal for the diagnosis of MS.^3 , 4^

During MS there are structural and functional changes in components of the vascular system.^5 - 7^ Individuals with MS present endothelial dysfunction, in addition to increased migration and proliferation of smooth muscle cells. An expansion of perivascular adipose tissue (PVAT) is also observed, as shown by morphological increase of adipocytes and by the replacement of brown adipose tissue with white adipose tissue, resulting in a reduction in the release of PVAT-derived relaxing factors and consequent lack of anti-contractile capacity.^8 - 11^

Hypertrophy and replacement of adipocytes in the PVAT may favor the production and accumulation of pro-inflammatory cytokines.^12^ Rodents that underwent a high-calorie diet and developed MS showed increase in and accumulation of PVAT in the aorta and the carotid arteries. This fact promoted the release of chemokines and recruitment of monocytes and T-cells to PVAT.^13^ In this inflammatory environment, increased production of interleukin-6 (IL-6) and infiltrate of tumor necrosis factor-alpha (TNF-α) releasing macrophages may also be observed in the PVAT.^14^ This set triggers chronic vascular system inflammation, impairing the structure and the function of the constituents of this system.

Blood vessel inflammation indeed contributes to vascular remodeling, increased peripheral resistance, and circulatory desorders.^7 , 15 , 16^ In this context, vascular remodeling is a chronic adaptive process characterized by changes in blood vessel structure derived from pro-inflammatory cytokines, in addition to growth factors, hemodynamic stimulation, and reactive oxygen species. It encompasses changes in cell growth, death, and migration and in synthesis and degradation of the extracellular matrix.^17 - 19^

An important factor to be considered in the vascular changes found during MS is dyslipidemia, a condition characterized by increased circulating levels of cholesterol and triglycerides and reduced levels of HDL.^20^ Numerous studies established that dyslipidemia leads to an inflammatory vascular response, reflected in the activation of endothelial cells, recruitment of leukocytes, and production of pro-inflammatory cytokines.^21 , 22^ Hypercholesterolemic mice fed with a high-fat diet showed remodeling in the medial layer of the femoral artery, associated with recruitment of macrophages.^23^ In this context, there is a positive correlation between levels of circulating cholesterol, chronic inflammation, and vascular remodeling.

Atorvastatin, an inhibitor of β-hydroxy-β-methylglutaryl-coenzyme A reductase, has shown to be one of the most effective statins in the treatment of dyslipidemias, reducing the production of LDL-cholesterol and increasing the production of high density lipoprotein (HDL) cholesterol.^24 , 25^ However, over the last decades, a growing body of evidence, both experimental and clinical, has accumulated to support the idea that atorvastatin exerts beneficial cardiovascular effects, regardless of its primary effects.^26^ In an experimental MS model, atorvastatin was able to improve reactivity and reduce structural remodeling of resistance arteries, both of which were associated with reduced inflammation and reduced oxidative stress.^27^ Furthermore, atorvastatin is able to inhibit the secretion of matrix metalloproteinase 9 in vascular cells and suppress the expression of transforming growth factor beta, thus reducing vascular fibrosis.^28 - 30^ These *in vitro* effects of atorvastatin also associated with its anti-inflammatory capacity.^31^ In the clinical context, atorvastatin reduces atherosclerotic processes^32^ and clinical manifestations of acute coronary syndrome.^33^ Indeed, one of the several pleiotropic effects of atorvastatin is its robust vasoprotective capacity. Nevertheless, there is no sufficient evidence to confirm the effects and the possible mechanisms involved in the actions of atorvastatin from the perspective of the vascular smooth muscle and, unprecedentedly, of PVAT as inflammatory tissues in conditions of MS. Therefore, in this study we hypothesize that MS leads to vascular remodeling of vascular smooth muscle and of PVAT, resulting from increased vascular inflammation. Moreover, atorvastatin therapy may be able to reduce vascular inflammation and thus revert the damages associated with MS.

## Methods

### Animals and experimental design

All experimental procedures were analyzed by the Ethics Committee on Animal Use of Universidade Federal de Jataí (Protocol 02/2019). Three-week old male Swiss mice were obtained from the Central Vivarium of Universidade Federal de Goiás – Regional Goiânia and housed in the Animal Experimentation Vivarium of Universidade Federal de Jataí, under a controlled temperature of 22 ± 2ºC, humidity of 50 ± 5%, and 12-hour light/dark cycles, and ad libitum access to water and food. During the entire experimental period, mice were kept in polypropylene cages (30 cm long x 20 cm wide x 13 cm high), in groups of 3 mice per cage.

After one week of acclimatization, mice received a control diet or a hyperglycemic diet for 18 weeks. After 14 weeks of diet, mice were treated (daily in the afternoon) by gavage with vehicle (saline) or atorvastatin (20mg/kg, Sigma-Aldrich, #PZ0001, Germany) for 4 weeks.^34^ The control diet consisted of 22% of protein, 70% of carbohydrate, and 8% of fat, whereas the hyperglycemic diet consisted of 10% of protein, 80% of carbohydrate, and 10% of fat. The hyperglycemic diet included 33% of control diet (Nuvilab® CR1, Nuvital, Brazil), 33% of condensed milk, and 7% of sucrose by weight, the remaining being water.^6^ The energy density was 12.16 kJ/g for the control diet and 13.35 kJ/g for the hyperglycemic diet. Mice were euthanized by overdose anesthesia (ketamine and xylazine, 140 mg/kg and 12 mg/kg, respectively, administered intraperitoneally).

### Nutritional and murinometric profile of the experimental model

The nutritional profile was determined by food consumption and consequent caloric intake, in addition to feed efficiency. Caloric intake (by mouse) was calculated based on weekly food intake multiplied by the energy content of the diet (g x kcal). In order to analyze the capacity of each mouse to convert the ingested calories into body mass, feed efficiency was calculated by dividing the overall body mass gain (g) by the overall energy intake (kcal), in percent. The murinometric profile was determined by analysis of body mass and fat. Mice’s body mass was measured every week using a high accuracy analytical balance, and obesity was characterized by the adiposity index {[body fat (g)/ final body weight (g)]x100} at the end of the diets and treatments, and body fat was calculated by the sum of epididymal, retroperitoneal and visceral fats.^35^

### Collection of serum and PVAT

Mice were subjected to an 8-hour fasting. After euthanasia, blood samples were collected through cardiac puncture and transferred into dry tubes. Blood was centrifuged at 2,500 rpm for 15 minutes to separate the serum to be used in the determination of mice’s biochemical and cytokine levels. PVAT was mechanically removed from segments of the thoracic aorta, frozen in liquid nitrogen, pulverized and homogenized in cold phosphate buffer solution (PBS). The tissue specimen was centrifuged at 13,000 rpm for 20 minutes to separate the insoluble material. The supernatant was collected for determination of cytokine levels.

### Assessment of lipid and glucose profiles

Serum levels of total cholesterol total, triglycerides, and HDL-cholesterol were determined by enzymatic methods (kits Labtest, Brazil) in blood samples obtained after whole blood centrifugation. The concentration of very low-density lipoproteins (VLDL) was calculated based on the serum concentration of triglycerides, using the following formula: VLDL (mg/dL)= Triglycerides (mg/dL)/5. The concentration of low-density lipoproteins (LDL) was calculated based on HDL and VLDL concentrations, using the following formula: LDL (mg/dL) = Total cholesterol total - HDL - VLDL.^36^ Glucose was determined after treatment with the diets. A drop of blood was applied to individual test strips specific for blood glucose measurement (Accu-Check Active, Germany).

### Oral glucose tolerance test (OGTT)

On the day before the end of the 18th week of diets and treatments, after 8-hour fasting, mice’s blood glucose was measured by a glucometer (Accu-Check Active, Germany), in order to determine baseline blood glucose levels. Subsequently, a 2 g/kg dose of glucose was administered to mice by gavage. From this moment on, a chronometer was started and new blood glucose measurements were conducted at 30, 60, 90 and 120 minutes.^37^

### Morphostructural assessment of vascular smooth muscle and PVAT

Specimens of thoracic aortas were removed and then fixed in 4% paraformaldehyde for 24 hours. The next stages involved dehydration in increasing concentrations of ethyl alcohol (70%, 80%, 90% and 100%) for 120 minutes, with subsequent diaphanization in absolute ethyl alcohol (1:1) 1, 2 and 3 for 120 minutes and in xylol 1, 2 and 3 for 30 minutes, with subsequent inclusion in paraffin. Aorta specimens were included in paraffin and sectioned in a microtome at a thickness of 4.5 µm for subsequent staining.

For hematoxylin and eosin staining, slides were deparaffinized, kept in a greenhouse at 65ºC, and then immersed in xylol 1 and 2 for 20 minutes. Subsequently, slides were immersed in decreasing concentrations of ethyl alcohol (100%, 90%, 70% and 50%) for 5 minutes and immersed in distilled water for 10 minutes. After hydration, slides were immersed in hematoxylin for 6 minutes and immediately after washed in running water for 10 minutes. Subsequently, they were immerse in eosin for 6 minutes. Sections were dehydrated and diaphanized again and then mounted with Permount® Mounting Medium (Fisher Scientific, USA) and glass slides.

Stained images were obtained by a digital camera coupled to an optical microscope and analyzed with the ImageJ software (National Institutes of Health, USA). For the analysis of images, lumen, tunica media, and tunica adventitia were delimited in order to calculate the following morphometric variables: cross-sectional area (CSA) and thickness of tunica media. The volume of adipocytes (v) were calculated by the formula v=πd^3^/6, where (d) represents the diameter of adipocytes. The mass of adipocytes was calculated by volume multiplied by density (0.92 g/cm^3^). The number of adipocytes was determined by dividing the mass of PVAT by mean mass of adipocytes.^38^

### Determination of levels of inflammatory cytokines

The levels of TNF-α and IL-6 in serum and in the PVAT were assessed by the immunoenzyme assay (ELISA) using DuoSet ELISA Development Systems kits (R&D Systems, USA) according to manufacturer’s instructions. Microtiter 96-well plates were covered with 50 μl/well of specific anti-TNF-α and anti-IL-6 antibodies at the concentrations described by the manufacturer, diluted in PBS, and incubated overnight at 4ºC. Plates were washed with PBS/Tween-20 (0.05%), and nonspecific binding sites were blocked with 100 μl of PBS containing 1% bovine serum albumin for 2 hours at room temperature. Subsequently, samples were added, and a new incubation was conducted for 2 hours at room temperature. After this period, plates were washed and 50μl of biotinylated antibodies specific for each cytokine were added. After 2 hours, plates were washed, and streptavidin-peroxidase conjugate diluted at 1:40 was added to each well. Plates were incubated for 1 hour at room temperature. Subsequently, plates were washed and 100μl of tetramethylbenzidine substrate were added. Optical density was measured at 630 nm in a SpectraMAX 190 Microplate Reader spectrophotometer (Molecular Devices, USA). The levels of cytokines in the samples were calculated based on an 11-point standard curve obtained by serial dilution. Results were expressed in pg/mL for serum samples and pg/mg of protein for samples of PVAT.

### Statistical analysis

Results were expressed as mean ± standard deviation (SD). Data followed a normal distribution, according to the Kolmogorov-Smirnov test. Results were analyzed by two-way analysis of variance (ANOVA), followed by Tukey post-hoc test. The minimum acceptable level of significance was p<0.05. For convenience reasons, the number of mice for each experimental group was 6. The Prisma software, version 8.0 (GraphPad Software Inc., USA) was used to analyze these parameters and to design the graphs.

## Results

### Nutritional and murinometric profile

Food intake and, consequently, calorie intake were higher in mice fed with the hyperglycemic diet, as well as feed efficiency. However, this last parameter was reduced after atorvastatin therapy. Murinometric parameters, such as mass gain, deposits of epididymal, retroperitoneal and visceral fats were higher in mice that received the hyperglycemic diet compared with those that received the control diet. Atorvastatin therapy led to a decrease in these parameters ( Table 1 ). Mice on the hyperglycemic diet showed a greater increase in body mass compared to those on the control diet. Furthermore, mice fed with the hyperglycemic diet and treated with atorvastatin had a decrease in their body mass ( Figure 1A ). The adiposity index, the parameter used to determine obesity, was higher in mice that received the hyperglycemic diet compared to those that received the control diet. Atorvastatin therapy led to a decrease in the adiposity index among mice on the hyperglycemic diet ( Figure 1B ).

**Table 1:** – Eating behavior and weight assessment of body fat deposits

	CD + Vehicle	CD + Atorvastatin	HD + Vehicle	HD + Atorvastatin
Food intake (g/week)	22.6 ± 0.3	21.3 ± 0.4	31.7 ± 0.2	31.1 ± 0.5
Calorie intake (kcal/week)	274.8 ± 3.7	259.1 ± 4.9	423.1 ± 2.67*	415.2 ± 6.7
Feed efficiency (%)	0.51 ± 0.01	0.53 ± 0.01	0.64 ± 0.02*	0.47 ± 0.03^#^
Mass gain (g)	25.0 ± 2.31	24.5 ± 2.25	37.3 ± 1.52*	27.9 ± 1.22^#^
Epididymal fat (g)	0.97 ± 0.12	1.13 ± 0.11	2.68 ± 0.24*	1.31 ± 0.09^#^
Retroperitoneal fat (g)	0.32 ± 0.06	0.61 ± 0.19	1.88 ± 0.12*	0.93 ± 0.19^#^
Visceral fat (g)	0.75 ± 0.15	0.94 ± 0.13	1.67 ± 0.11*	1.01 ± 0.17^#^

Dados expressed as mean ± SD. *, p<0.05 vs. CD + Vehicle;^#^, p<0.05 vs. HD + Vehicle (n=6 for each experimental group). CD: Control Diet, HD: Hyperglycemic Diet.

**Figure 1:** – Atorvastatin attenuates obesity associated with metabolic syndrome induced by hyperglycemic diet. The figures show body mass (A) and adiposity index (B) of mice fed with a control diet or with a hyperglycemic diet for 18 weeks and treated with vehicle solution or atorvastatin (20 mg/kg for 4 weeks). Results expressed as mean ± SD. *, p<0.05 vs. Control Diet_Vehicle; #, p<0.05 vs. Hyperglycemic Diet_Vehicle (n=6 for each experimental group).

### Lipid profile

Mice fed with the hyperglycemic diet had dyslipidemia compared with those fed with the control diet. Dyslipidemia may be observed based on increased levels of total cholesterol ( Figure 2A ), in which LDL ( Figure 2B ) and VLDL ( Figure 2C ) fractions were increased and HDL fraction ( Figure 2D ) was reduced. There was also an increase in the levels of triglycerides of mice that received the hyperglycemic diet compared to those that received the control diet ( Figure 2E ). Mice on the hyperglycemic diet and treated with atorvastatin showed a decrease in these lipid parameters.

**Figure 2:** – Atorvastatin attenuates dyslipidemia associated with metabolic syndrome induced by hyperglycemic diet. The figures show the levels of total cholesterol (A), LDL (B), VLDL (C), HDL (D), and triglycerides (E) of mice fed with a control diet or with a hyperglycemic diet for 18 weeks and treated with vehicle solution or atorvastatin (20 mg/kg for 4 weeks). Results expressed in mean ± SD. *, p<0.05 vs. Control Diet_Vehicle; #, p<0.05 vs. Hyperglycemic Diet_Vehicle (n=6 for each experimental group).

### Glucose profile

Fasting serum glucose of mice fed with the hyperglycemic diet was increased when compared to that of mice fed with the control diet. Mice that received the hyperglycemic diet and atorvastatin therapy did not show a decrease in glucose concentration ( Figure 3A ). In the OGTT ( Figure 3B ), mice on the hyperglycemic diet had an increased glucose concentration compared with mice on the control diet, and blood glucose levels did not return to baseline levels over time, as observed in mice on control diet. Moreover, mice fed with the hyperglycemic diet and treated with atorvastatin had lower blood glucose levels over time. The area under the curve shows the above mentioned effects ( Figure 3C ).

**Figure 3:** – Atorvastatin attenuates glucose intolerance associated with metabolic syndrome induced by hyperglycemic diet. The figures show the levels of fasting glucose (A), glucose curves (B), and area under the curve (C) obtained in the oral glucose tolerance tests of mice fed with a control diet or with a hyperglycemic diet for 18 weeks and treated with vehicle solution or atorvastatin (20 mg/kg for 4 weeks). Results expressed as mean ± SD. *, p<0.05 vs. Control Diet_Vehicle; &, p<0.05 vs. Control Diet_Atorvastatin; #, p<0.05 vs. Hyperglycemic Diet_Vehicle (n=6 for each experimental group).

### Morphology of smooth muscle

Mice fed with the hyperglycemic diet had an increase in vascular CSA compared to those fed with the control diet. Mice that received the hyperglycemic diet and were treated with atorvastatin showed decreased CSA ( Figure 4A ). Similarly, there was an increase in the thickness of vessel tunica media among mice on the hyperglycemic diet compared to those on the control diet. Atorvastatin therapy was able to reduce this increase in thickness of tunica media ( Figure 4B ).

**Figure 4:** – Atorvastatin attenuates remodeling of vascular smooth muscle associated with metabolic syndrome induced by hyperglycemic diet. The figures show representatives images, size of CSA (A) and vessel wall thickness (B) of mice fed with a control diet or with a hyperglycemic diet for 18 weeks and treated with vehicle solution or atorvastatin (20 mg/kg for 4 weeks). Results expressed as mean ± SD. *, p<0.05 vs. Control Diet_Vehicle; #, p<0.05 vs. Hyperglycemic Diet_Vehicle (n=6 for each experimental group). CD: Control Diet, HD: Hyperglycemic Diet.

### Morphology of perivascular adipose tissue

In order to investigate whether there were changes in PVAT morphology, the present study assessed the size and the number of adipocytes presents in the tunica adventitia. It was possible to observe that mice fed with the hyperglycemic diet had larger adipocytes ( Figure 5A ) and in a smaller number ( Figure 5B ) compared to mice fed with the control diet. The group that received the hyperglycemic diet and atorvastatin therapy showed a reduction in the size of adipocytes and an increase in their number.

**Figure 5:** – Atorvastatin attenuates PVAT remodeling associated with metabolic syndrome induced by hyperglycemic diet. The figures show representative images (A) and size of PVAT adipocytes (B) of mice fed with a control diet or with a hyperglycemic diet for 18 weeks and treated with vehicle solution or atorvastatin (20 mg/kg for 4 weeks). Results expressed as mean ± SD. *, p<0.05 vs. Control Diet_Vehicle; #, p<0.05 vs. Hyperglycemic Diet_Vehicle (n=6 for each experimental group). CD: Control Diet, HD: Hyperglycemic Diet.

### Inflammatory profile

Concentrations of the pro-inflammatory cytokines called TNF-α ( Figures 6A and 6C ) and IL-6 ( Figures 6B and 6D ) were determined in the serum and in PVAT. Mice on the hyperglycemic diet showed higher concentration of TNF-α and IL-6, both in serum and in PVAT, compared to mice on the control diet. Atorvastatin therapy reduced the levels of TNF-α and IL-6 both in the serum and in the PVAT of mice fed with the hyperglycemic diet.

**Figure 6:** – Atorvastatin attenuates increased concentrations of pro-inflammatory cytokines associated with metabolic syndrome induced by hyperglycemic diet. The figures show the levels of TNF-α (A and C) and IL-6 (B and D) in serum and in PVAT, respectively, of mice fed with a control diet or with a hyperglycemic diet for 18 weeks and treated with vehicle solution or atorvastatin (20 mg/kg for 4 weeks). Results expressed as mean ± SD. *, p<0.05 vs. Control Diet_Vehicle; &, p<0.05 vs. Control Diet_Atorvastatin; #, p<0.05 vs. Hyperglycemic Diet_Vehicle (n=6 for each experimental group).

## Discussion

The hyperglycemic diet resulted in murinometric and metabolic changes in mice. Silva et al.^39^ subjected an experimental group to a similar diet and found increases in parameters identical to those used in this study.^39^ In this context, Leopoldo et al.,^40^ using experimental models, defined the categories of adiposity after study participants received a hyperglycemic diet, which vary from: normal – adiposity index (%) 4.17-4.55; overweight – adiposity index (%) 5.69-6.23; and obesity – adiposity index (%) 7.53-10.02.^40^ Our results showed an adiposity index of nearly 10% for mice fed with the hyperglycemic diet, which classifies the experimental group as obese.

Glucose is a carbohydrate that represents one of the main energy sources for the body and, when in excess, through metabolic pathways, is stored as glycogen. If the capacity to store carbohydrate intake exceeds glycogenesis, the remaining excess is converted into triacylglycerol and incorporated into cholesterols obtained from the diet and into apolipoproteins, responsible to transport triglycerides to storage tissues, such as adipose tissue.^41^ Thus, carbohydrate excess in the diet of mice contributes to increases in body mass and adiposity index.

Mice that received the hyperglycemic diet showed dyslipidemia and increased fasting serum glucose. Mullugeta et al.^42^ showed that hyperglycemia and insulin resistance are associated with dyslipidemia in individuals with type II diabetes mellitus, i.e., hyperglycemia leads to impaired lipid metabolism. Furthermore, it was found that hyperglycemic individuals had increased serum levels of triglycerides, total cholesterol and its fractions compared to normoglycemic individuals.^42 , 43^

Atorvastatin therapy proved to be efficient to control the levels of total cholesterol and its fractions. Moreover, atorvastatin reduced the levels of triglycerides, body mass, and adiposity index. Silva et al.^44^ also showed the effectiveness of atorvastatin in daily doses of 10 mg or 20 mg on the correction of individuals’ lipid profile, in which cholesterol therapy led also to a decrease in the level of triglycerides.^44^ It is known that VLDL and LDL are lipoproteins consisting significant amounts of triacylglycerols; thus, we propose that by reducing the levels of VLDL and LDL through atorvastatin therapy may lead to reduced levels of triglycerides. Parhofer et al.^45^ describes the lowering effect of triglycerides, especially by the inhibition of cholesterol synthesis, and also highlight that VLDL and LDL have the same removal mechanism; hence, the reduction in the number of circulating LDL particles may also significantly reduce VLDL levels.^45^

According to the OGTT, the hyperglycemic diet induced glucose intolerance in mice. Chronic exposure to hyperglycemia is known to eventually affect insulin activity with regard to synthesis, secretion of action of this hormone, which is indeed observed in type II diabetes mellitus.^6^ Therefore, it is possible to suggest that the results found for the glucose profile of mice on the hyperglycemic diet contribute to the development of type II diabetes mellitus. Kissebah et al.^46^ showed that increased visceral fat is associated with glucose intolerance and insulin resistance, and Taylor et al.^47^ found that visceral fat is related to the development of diabetes mellitus.^46 , 47^ Previous studies described that increased visceral fat predisposes to production and secretion of cytokines, such as IL-6 and TNF-α, and adipokines, such as resistin and adiponectin, which are associated with the development of insulin resistance and disorders in glucose metabolism and control, due to interference with insulin intracellular signaling.^48 , 49^ Increased visceral fat, as well and increased levels of TNF-α and IL-6, justify the imbalances in glucose profile.

Atorvastatin therapy was able to attenuate glucose intolerance in mice fed with the hyperglycemic diet. Huptas et al.^50^ obtained promising results with atorvastatin therapy in individuals with MS, showing improvements in glucose metabolism through reduced glucose intolerance and reduced insulin resistance. Using experimental models of glucose intolerance and insulin resistance, Suzuki et al.^51^ performed OGTT and insulin tolerance test (ITT) in groups treated and not treated with atorvastatin and showed that the treated group had reduced levels of glucose and insulin in OGTT and ITT compared to the group that received the vehicle solution.^50 , 51^

The mechanisms through which atorvastatin improves parameters related to glucose metabolism are not known yet. This improvement may be related to decreased gluconeogenesis.^51^ However, it is possible to suggest that reduced glucose intolerance may be related to reduced deposits of visceral fat and may also be related to reduced production of cytokines (TNF-α and IL-6), which are potential causes of metabolic disorders.

According to Mulvany (1999), vascular remodeling may be classified based on measures of CTA and wall thickness. Increased CTA and wall thickness is typical of hypertrophic remodeling.^52^ One of the causes of vascular remodeling is inflammation mediated by increased levels of pro-inflammatory cytokines. Studies on MS showed that PVAT hypertrophy results in migration, activation of immune cells and secretion of the above mentioned cytokines, leading to a low-intensity chronic inflammatory process.^53 - 55^

Changes in the structure and composition of PVAT reflect in vessel tunica media, because PVAT is physiologically constituted by adipocytes that act as regulators of the proliferation of vascular smooth muscle cells (VSMCs). The change to non-specialized adipocytes culminates in increased VSMC proliferation and smooth muscle hypertrophy. This hypertrophy has a major role in vascular diseases such as restenosis and arterial hypertension.^56^

TNF-α and IL-6 are found in increased concentrations during chronic inflammation present in obesity, insulin resistance, and MS. Additionally, there is a clear relationship between increased levels of these cytokines and phenotypic changes in PVAT.^57 , 58^ Pro-inflammatory cytokines promote vascular remodeling and dysfunction, since increased concentrations of TNF-α promote neointimal hyperplasia and endothelial dysfunction. Similarly, increased concentration of IL-6 may induce macrophage infiltration into PVAT, contributing to development of aneurism and vascular remodeling associated with PVAT inflammation.^59 , 60^

Hypercholesterolemia was induced from a hyperlipidemic diet in rabbits to investigate what changes this condition could develop in their pulmonary arteries. These arteries had increased VSMC proliferation, tunica media hypertrophy, and tunica intima hyperplasia. Furthermore, there were a large infiltrate of inflammatory cells, e.g., macrophages, in lung tissue a higher concentration of serum IL-6 in the rabbits. These animals were treated with atorvastatin, which led to a reversal of hypercholesterolemia, vascular remodeling, and inflammatory processes in lung tissue.^61^ Similarly, atorvastatin inhibits aldosterone-induced vascular remodeling by decreasing pro-inflammatory cytokines.^31^

The main limitation of this study was the impossibility of establishing a direct causal relationship of pharmacological therapy with atorvastatin with improvements in metabolic profile and vascular remodeling. Based on the experimental design used in this study, we showed that atorvastatin reduces adiposity and improves lipid, glucose and inflammatory profiles. Literature has extensively shown that these facts are beneficial for vascular structure and function. Therefore, it is not clear yet whether attenuated vascular remodeling may be explained by direct actions of atorvastatin on vessels or by effects underlying the metabolism. We reinforce that, in multicomplex organisms, isolated mechanisms do not justify the genesis of diseases, since it always involves a vast interaction between mechanisms and systems. Therefore, regardless whether atorvastatin has a direct or pleiotropic action, the final therapy has shown to be promising for the context of this study.

## Conclusions

In short, this study shows that atorvastatin therapy attenuates the metabolic damages associated with MS induced by hyperglycemic diet, in addition to attenuating remodeling of vascular smooth tissue and PVAT; these effects are associated with reduced levels of the pro-inflammatory cytokines TNF-α and IL-6.
